# Effects of exercise training on patients with lung cancer who underwent lung resection: a meta-analysis

**DOI:** 10.1186/s12957-017-1233-1

**Published:** 2017-08-23

**Authors:** Jie Li, Nan-Nan Guo, Hai-Rong Jin, Hua Yu, Peng Wang, Guo-Gang Xu

**Affiliations:** 1 Department of Thoracic Surgery, Chinese PLA General Hospital, Beijing, 100853 China; 2 Nanlou Medical Oncology Department, Chinese PLA General Hospital, Beijing, 100853 China; 3 Nanlou Respiratory Diseases Department, Chinese PLA General Hospital, No. 28 Fuxing Road, Haidian District, Beijing, 100853 China

**Keywords:** Lung cancer, Exercise, Quality of life, Meta-analysis

## Abstract

**Background:**

The efficacy of exercise training in patients with lung cancer after lung resection has not been well established yet. Therefore, we performed a meta-analysis to investigate the efficiency of exercise training in patients with lung cancer after lung resection.

**Methods:**

Several databases were searched for eligible randomised controlled trials (RCTs). The primary outcome was quality of life, and the secondary outcomes included 6-min walk distance (6MWD), forced expiratory volume in 1 s (FEV_1_) and postoperative complications (POCs). Weighted mean differences (WMDs) and relative risks (RRs) with 95% confidence intervals (CIs) were calculated by random-effects model.

**Results:**

Six RCTs involving 438 patients were enrolled in this meta-analysis. The pooled WMDs of the scores were 2.41 (95% CI = −5.20 to 10.02; *P* = 0.54) and −0.46 (95% CI = −20.52 to 19.61; *P* = 0.96) for the physical and mental components of the 36-item short-form scale, respectively. The pooled WMDs were 23.50 m (95% CI = −22.04 to 69.03; *P* = 0.31) for 6MWD and 0.03 L (95% CI = −0.19 to 0.26; *P* = 0.76) for FEV_1_. Finally, the pooled RRs were 0.79 (95% CI = 0.41 to 1.53; *P* = 0.49) for POCs.

**Conclusions:**

Insufficient evidence is available to support the efficacy of exercise training in patients with lung cancer after lung resection. Further studies must confirm our findings and investigate the long-term effects of exercise training on patients with lung cancer following lung resection.

**Electronic supplementary material:**

The online version of this article (doi:10.1186/s12957-017-1233-1) contains supplementary material, which is available to authorized users.

## Background

Cancer is an important public health problem worldwide, and lung cancer accounts for more than one-quarter (27%) of all deaths related to cancer [1]. Lung resection is the most effective treatment approach for patients with lung cancer, especially for those with early-stage non-small cell lung cancer (NSCLC) [2]. However, patients who underwent lung resection tend to experience deteriorated exercise capacity, lung function and quality of life (QoL); moreover, these patients commonly experience various cancer-related complications, including postoperative complications (POCs), dyspnoea, pain, fatigue and loss of appetite [3–7]. A multidisciplinary approach has been increasingly investigated for appropriate management of patients with lung cancer. Pulmonary rehabilitation (PR) is an effective treatment not only for chronic obstructive pulmonary disease (COPD) but also for several respiratory conditions, such as asthma, cystic fibrosis, lung transplantation and lung cancer [8–13].

Scholars have proposed that PR programs, including walking [14], exercise training [15], inspiratory muscle training [16], respiratory physiological adaptability training [17] and Tai Chi [18], can improve pulmonary and physical function, decrease the risk of POCs and the length of hospital admission and potentiate human immunity against tumours. These programs can also be used to manage patients with lung cancer [19]. Several published randomised controlled trials (RCTs) [15, 16, 20–22] and non-RCTs [23–25] evaluated the role of PR in patients with lung cancer after lung resection. However, these trials were initially designed to compare different primary endpoints due to different foci; moreover, clinically important endpoints, such as exercise capacity and QoL, have not been adequately investigated due to limited data in each trial. Results of these trials are inconclusive because of the wide variation in sample sizes employed. Thus far, the effect of exercise training on patients with lung cancer after lung resection remains controversial. In the present study, we performed a meta-analysis on available RCTs to investigate the role of exercise training in adult patients following lung cancer surgery.

## Methods

### Data sources and selection criteria

Several databases including PubMed, Cochrane, CINAHL, EMBASE and PEDro were searched for eligible RCTs up to February 2017. The search strategies for PubMed are presented in Additional file 1: Table S1 and were used for the other databases. No language restriction was implemented. The search was restricted to adult subjects. To ensure data saturation, we manually searched the reference lists of included studies for unpublished studies and reviews to identify any potentially eligible trials.

The available RCTs were selected with the following criteria: (i) population: adult patients with lung cancer who underwent lung resection, (ii) intervention: various forms of exercise trainings, including endurance, resistance, strength, treadmill and walking, (iii) control: usual care or standard postoperative care, (iv) outcomes: the primary outcome was QoL, and the secondary outcomes included 6-min walk distance (6MWD), forced expiratory volume in 1 s (FEV_1_) and POCs and (v) study design: randomised controlled trial.

### Data extraction and outcome measurement

Two authors independently extracted the following data from the studies: first author; publication year; sample size per group (intervention/control); age; protocol of exercise training (e.g. exercise type, time per session, frequency, intensity and duration); outcomes; study designation and Jadad scale. Disagreements were resolved by a third author. In addition, analytical data missing from the original published studies were requested from the respective authors.

The predefined primary outcome was QoL, and the secondary outcomes included 6MWD, FEV_1_ and POCs. The QoL evaluation scales included the Medical Outcome Study 36-item Short-Form Health Survey (SF-36) [26], the European Organisation for Research and Treatment of Cancer Core Quality of Life Questionnaire 30 (EORTC QLQ-C30) [27] and St. George’s Respiratory Questionnaire (SGRQ) [28]. Considering the limited QoL data, we conducted the meta-analysis of the physical and mental component scores only of the SF-36 scale; high scores indicate better QoL. POCs were defined as X-ray changes reported by the radiologist; POCs include pneumonia, respiratory complications requiring additional ventilatory support and return to high-dependency care and death and transfer to critical care > 72 h after the surgery [15, 16, 20].

### Quality and risk-of-bias assessment

The methodological quality was evaluated according to the Jadad scale [29]. In detail, randomisation (0–2 points), blinding (0–2 points) and dropouts and withdrawals (0–1 point) were identified in the scale. A trial with a score ≤ 2 indicates low quality, and that with a score of ≥ 3 indicates high quality [30]. In addition, the risk of bias was assessed by the Cochrane Risk of Bias Assessment Tool [31]. A third author (GGX) resolved any disagreements regarding classification of study quality components.

### Statistical analysis

This meta-analysis was conducted in accordance with the Preferred Reporting Items for Systematic Reviews and Meta-Analyses statement [32]. Weighted mean differences (WMDs) and relative risks (RRs) with 95% confidence intervals (CIs) for continuous and dichotomous outcomes were calculated by random-effects model [33]. Heterogeneity was tested using Cochrane’s *Q* test and *I* ^2^ statistic, where *I* ^2^ > 50% was classified as significant heterogeneity [34]. Furthermore, sensitivity analyses were conducted to explore the potential sources of heterogeneity and investigate the influence of a single study on the overall pooled estimate. Potential publication bias was evaluated using funnel plots. All data and statistical analyses were performed using RevMan 5.3 (the Cochrane Collaboration, Oxford, UK). Finally, a two-sided *P* < 0.05 indicated statistical significance, and the overall results were compared with the minimum clinically important difference (MCID).

## Results

### Eligible studies

**Fig. 1:**
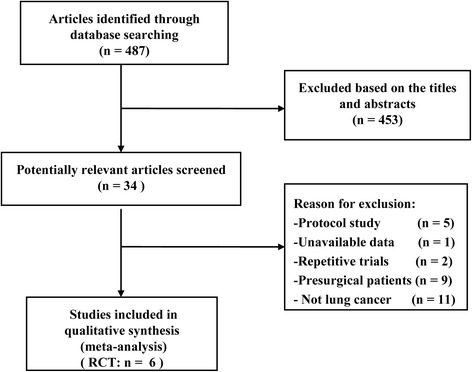
Search strategy and flow chart (randomised controlled trials; RCTs)

Initially, 487 potential studies were retrieved from the computerised electronic search. Based on titles and abstracts, 453 studies were excluded because they are unrelated to the aims of the present work. Twenty-eight candidate studies were further excluded for various reasons (Fig. 1). Finally, six RCTs were selected for the meta-analysis [15, 16, 20–22, 35]. Only one of these RCTs failed to be included for full-text analysis [35].

### Study characteristics

Table 1 summarises the main characteristics of the six RCTs, with a total of 438 patients. All RCTs were made available in English and conducted between 2010 and 2015. The sample size of all trials ranged from 49 to 131. Five RCTs [15, 20–22, 35] included patients with NSCLC only [16]. The duration of exercise training ranged from 2 to 20 weeks, and the exercise lasted for 5–60 min per session. Two RCTs [16, 35] did not report the exact exercise duration per session. All of the RCTs included applied different forms and intensities of exercise.

**Table 1:** Characteristics of randomised controlled trials included in the meta-analysis

Study/year	Patients no. (I/C)	Cancer type	Age, mean, years (I/C)	Intervention group					Control group	Primary outcome	Secondary outcomes	Study design/Jadad score
				Type of PR	Time/session	Frequency	Intensity	Duration				
Arbane et al., [15]	51 (26/25)	NSCLC	65.4/62.6	Strength and mobility training	5–10 min	Twice daily	60–80% MHR	12 weeks + 5 days	Usual care	6MWD	POC, QoL, quadriceps strength	RCT/4
Arbane et al., [20]	131 (64/67)	NSCLC	67/68	Hospital plus home exercise	30 min	Once daily	60–90% MHR	4 weeks	Usual care	Physical activity	POC, QoL, quadriceps strength	RCT/4
Brocki et al., [43]	78 (41/37)	NSCLC	64/65	Aerobic exercise + resistance training + dyspnoea management	NA	NA	60–80% peak work capacity	12 weeks	Usual care	QoL	6MWD, FEV_1_	RCT/3
Brocki et al., [16]	68 (34/34)	NSCLC + metastatic tumour + other type	69.7/70.5	Inspiratory muscle training	NA	Twice daily	30% of MIP	2 weeks	Standard physiotherapy treatment	Inspiratory muscle strength	6MWD, FEV_1_, dyspnoea, POC	RCT/4
Edvardsen et al., [22]	61 (30/31)	NSCLC	64.4/65.9	High-intensity endurance and strength training	60 min	Three times a week	80–95% MHR	20 weeks	Standard postoperative care	Peak oxygen uptake	FEV_1_, QoL, muscular strength and mass	RCT/4
Stigt et al., [21]	49 (23/26)	NSCLC	63.6/63.2	Aerobic (cycling) + resistance	60 min	Twice weekly	60–80% peak load	12 weeks	Usual care	QoL	6MWD, FEV_1_, pain	RCT/4

*I/C* intervention/control, *NSCLC* non-small cell lung cancer; *MHR* maximum heart rate, *6MWD* 6-min walk distance, *QoL* quality of life, *RCT* randomised controlled trial, *NA* not available, *FEV*_*1*_ the forced expiratory volume in 1 s, *MIP* maximal inspiratory pressure

### Quality and risk-of-bias assessment

**Fig. 2:**
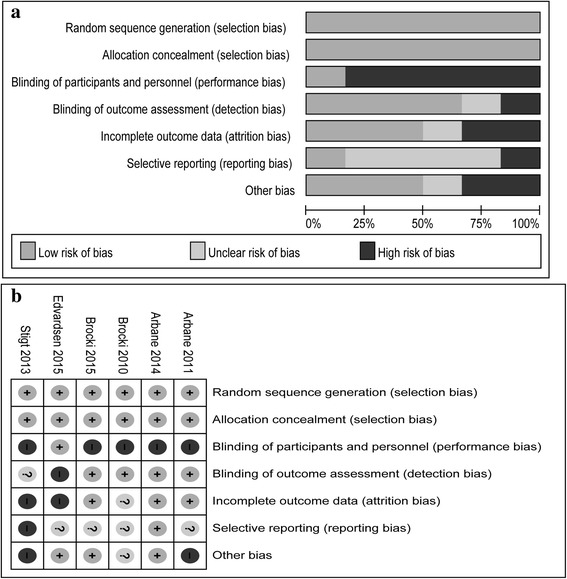
Risk-of-bias assessment: risk-of-bias graph (**a**) and risk-of-bias summary (**b**)

The mean Jadad score of all RCTs was 4.0 (SD = 0.6). The risk-of-bias assessment showed that all RCTs exhibited low risk in terms of random sequence generation and allocation concealment. Table 1 and Fig. 2 show the details of quality and risk-of-bias assessment, respectively.

### Meta-analysis of outcome measures

**Fig. 3:**
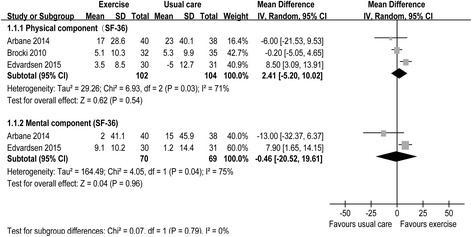
Forest plot of QoL, including the physical and mental components of the SF-36 scale

**Fig. 4:**
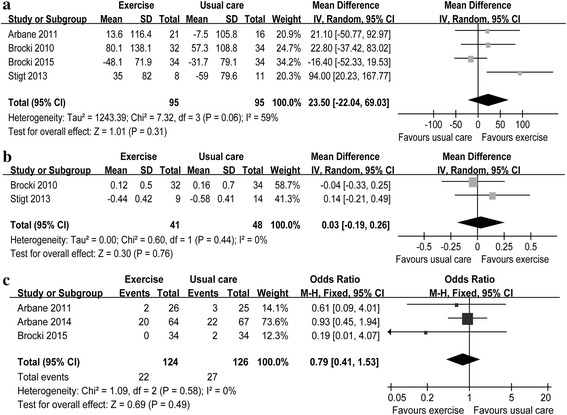
Forest plot of secondary outcomes including 6MWD (**a**), FEV_1_ (**b**) and POCs (**c**)

The pooled WMDs of the scores were 2.41 (three RCTs [20, 22, 35]; 95% CI = −5.20 to 10.02; *P* = 0.54; *P* for heterogeneity, 0.03; *I* ^2^ = 71%) and −0.46 (two RCTs [20, 22]; 95% CI = −20.52 to 19.61; *P* = 0.96; *P* for heterogeneity, 0.04; *I* ^2^ = 75%) for the physical and mental components of the SF-36 scale, respectively (Fig. 3). The pooled WMDs were 23.50 m (four RCTs [15, 16, 21, 35]; 95% CI = −22.04 to 69.03; *P* = 0.31; *P* for heterogeneity, 0.06; *I* ^2^ = 59%) for 6MWD (Fig. 4a) and 0.03 L (two RCTs [21, 35]; 95% CI = −0.19 to 0.26; *P* = 0.76; *P* for heterogeneity, 0.44; *I* ^2^ = 0%) for FEV_1_ (Fig. 4b). The pooled RRs were 0.79 (three RCTs [15, 16, 20]; 95% CI = 0.41 to 1.53; *P* = 0.49; *P* for heterogeneity, 0.58; *I* ^2^ = 0%) for POCs (Fig. 4c).

The physical component QoL exhibited high heterogeneity. We conducted sensitivity analyses to explore the potential source of heterogeneity for the physical component. The exclusion of the study conducted by Edvardsen et al. [22] resolved the heterogeneity but failed to change the results (WMD = −0.71 scores, 95% CI = −5.34 to 3.91; *P* = 0.76; *P* for heterogeneity, 0.48; *I* ^2^ = 0%). Further exclusion of the other trials did not resolve the heterogeneity and the results [(WMD = 4.06 scores, 95% CI = −4.46 to 12.59; *P* = 0.35; *P* for heterogeneity, 0.02; *I* ^2^ = 82%) [20], (WMD = 3.15 scores, 95% CI = −10.56 to 16.86; *P* = 0.65; *P* for heterogeneity, 0.08; *I* ^2^ = 67%) [35], respectively]. Considering that only two RCTs were left, we failed to perform sensitivity analyses to explore the potential source of heterogeneity for the mental component.

### Publication bias

Potential publication bias was evaluated using funnel plots when the sample size is small. Additional file 2: Figure S1 shows the types of publication bias for the primary outcome. The results from the analysis of the funnel plots showed no evidence of publication bias.

## Discussion

This study conducted comprehensive meta-analysis of available RCTs to evaluate the role of exercise training in adult patients with lung cancer who underwent lung resection. Eligible evidence suggested that exercise training program may be ineffective in improving QoL, exercise capacity and lung function and in decreasing the incidence of POCs. We believe that insufficient evidence is available to support the positive effects of exercise training on patients with lung cancer after lung resection.

Several systematic reviews have been published to describe the effects of exercise intervention on patients with NSCLC following lung resection [36–38]. The present findings show similarity and differences from previous reports. Cavalheri et al. [36, 37] conducted Cochrane systematic reviews of three RCTs with a total of 178 participants. By contrast, our meta-analysis included six RCTs with a total of 438 patients. Considering the limited data on the topic, we combined the latest three RCTs to increase the sample size, strengthen the test performance and produce robust results. In addition, we believe that the analysis of the pooled results may be unsuitable. The final values, instead of the within-group differences (i.e. the difference between baseline and post-intervention in the same group), after administering exercise intervention, were used to pool the outcomes, leading to increased risk of bias and unreliable results. Another narrative review did not conduct a meta-analysis [38]. Therefore, in contrast to aforementioned studies, we combined the three latest RCTs with a large sample size and applied changes from baseline and after intervention for meta-analysis of the outcomes of exercise intervention.

In this study, exercise training did not significantly improve QoL, 6MWD and FEV_1_ and did not decrease POCs. A significant heterogeneity was found during analysis of QoL. The exclusion of the study conducted by Edvardsen et al. [22] resolved the heterogeneity but failed to change the results. Further exclusion of the other trials did not resolve the heterogeneity and the results. Given the limited data, we could not define the probable sources of heterogeneity for QoL from various clinical characteristics (e.g. different exercise parameters). MCID was defined as the smallest difference considered significant by average patients and a recognised standard for determining the effectiveness of interventions in clinical trials [39]. Comparison of the pooled results included in our study with the MCID showed no statistically significant differences. No MCID is currently available for QoL evaluated by the SF-36 scale and for FEV_1_ and POCs in patients with lung cancer. Meanwhile, Granger CL [40] published an MCID for 6MWD in lung cancer, but this MCID was used to estimate deterioration rather than improvement. Further studies must be conducted to determine whether the MCID for deterioration is the same as that for improvement. Hence, the MCID reported should not be applied yet for determining improvement in patients with lung cancer. Osoba et al. suggested that changes in the 5–10 scores of EORTC QLQ-C30 represented a MCID in patients with lung cancer [41]. Only three of the RCTs included in the present meta-analysis reported QoL evaluated by the EORTC QLQ-C30 scale [15, 20, 22]. Of these three trials, one reported dyspnoea score [22], and the other trials did not provide related data [20]. Therefore, we could not pool the results for meta-analysis of QoL evaluated by EORTC QLQ-C30. Furthermore, three evaluation methods were used to assess QoL; such methods include SF-36, EORTC QLQ-C30 and SGRQ. The differences in the evaluation methods used complicate the assessment of QoL. Therefore, further studies are needed to determine an appropriate evaluation approach and a consistent evaluation scale for assessment of QoL and define MCID for patients with lung cancer who underwent lung resection.

This work presents valuable information for future clinical research on the effects of exercise training on patients with lung cancer after lung resection. Firstly, exercise programs included various forms, and no ‘standard’ was followed. A suitable form of exercise and appropriate training parameters has not been standardised yet for patients with lung cancer. Therefore, the optimal exercise prescriptions should be individualised based on patient characteristics. Additional studies must focus on establishing suitable forms of exercise for patients with lung cancer who underwent lung resection. Secondly, several studies suggested that Tai Chi may ameliorate the imbalance between humoral and cellular immunity and potentiate human immunity against tumours [18, 42]. Therefore, future research must focus on other exercise forms, such as Tai Chi and Yoga, in addition to general exercise trainings. Thirdly, most studies included in the meta-analysis lack other objective outcome measurements, such as peripheral muscle strength, overall survival and immune function, especially at the cellular and molecular levels. Further research should focus on the above-mentioned endpoints to obtain reliable and convincing evidence with regard to the effect of exercise training on patients with lung cancer who underwent lung resection. Finally, exercise training can benefit patients with COPD. Hence, patients with lung cancer, which is associated with COPD, may also benefit from exercise training. Further large-scale studies must be conducted to investigate the efficiency of exercise training in patients with lung cancer, especially for those with COPD.

Although this meta-analysis was not registered, the study was conducted in accordance with the PRISMA guidelines and the recommendations of the Cochrane Collaboration. Our results should be carefully interpreted, considering the following: (i) different exercise forms and parameters are probably the most crucial confounders, which contributed to a certain risk of bias and heterogeneity and influenced the overall results; (ii) few data are available (no more than two to four studies that reported outcomes), thereby influencing the interpretation of the results; and (iii) the primary outcome measurement is inconsistent among all trials, and QoL data were not obtained, resulting in possible selection bias.

## Conclusions

Insufficient evidence is available to support the efficacy of exercise training on patients with lung cancer after lung resection. Given the limitations and potential bias of our work, further large-scale robust studies must be conducted to confirm our findings and investigate the long-term effects of exercise training on this group of patients.

## Additional files

Additional file 1: **Table S1.** Search strategies for PubMed. (DOCX 11 kb)

Additional file 2: **Figure S1.** Publication bias. (TIFF 840 kb)

## Abbreviations

6MWD: 6-min walk distance
CI: Confidence interval
COPD: Chronic obstructive pulmonary disease
EORTC QLQ-C30: The European Organisation for Research and Treatment of Cancer Core Quality of Life Questionnaire 30
FEV_1_: Forced expiratory volume in 1 s
MCID: Minimum clinically important difference
NSCLC: Non-small cell lung cancer
POCs: Postoperative complications
PR: Pulmonary rehabilitation
QoL: Quality of life
RCT: Randomised controlled trial
RRs: Relative risks
SF-36: 36-item Short Form Health Survey
SGRQ: The St. George’s respiratory questionnaire
WMDs: Weighted mean differences
