# Supplementary material for: Effects of exercise training on patients with lung cancer who underwent lung resection: a meta-analysis
Source: World J Surg Oncol. 2017 Aug 23;15:158. doi: 10.1186/s12957-017-1233-1 (PMC5569526; doi:10.1186/s12957-017-1233-1)
Supplement: Supplementary file 1 — Table S1. Search strategies for PubMed. (DOCX 11 kb) [file 12957_2017_1233_MOESM1_ESM.docx]

| **Table S1. Search strategies for PubMed** | |
| --- | --- |
| #1 | ((((lung cancer*[Title/Abstract]) OR non-small cell [Title/Abstract]) OR non small cell [Title/Abstract]) OR Lung Neoplasms [MeSH]) OR Carcinoma, Non-Small-Cell Lung [MeSH] |
| #2 | (((((((((exercis*[Title/Abstract]) OR rehabilitat*[Title/Abstract]) OR aerobic [Title/Abstract]) OR endurance [Title/Abstract]) OR strength*[Title/Abstract]) OR inspiratory muscle*[Title/Abstract]) OR respiratory muscle*[Title/Abstract]) OR treadmill [Title/Abstract]) OR walking [Title/Abstract]) OR cycl*[Title/Abstract] #3 (training*[Title/Abstract]) |
| #3 | (training*[Title/Abstract]) |
| #4 | ((#1) AND #2) AND #3 |
