# Supplementary figures and images for: Effects of exercise training on patients with lung cancer who underwent lung resection: a meta-analysis
Source: World J Surg Oncol. 2017 Aug 23;15:158. doi: 10.1186/s12957-017-1233-1 (PMC5569526; doi:10.1186/s12957-017-1233-1)

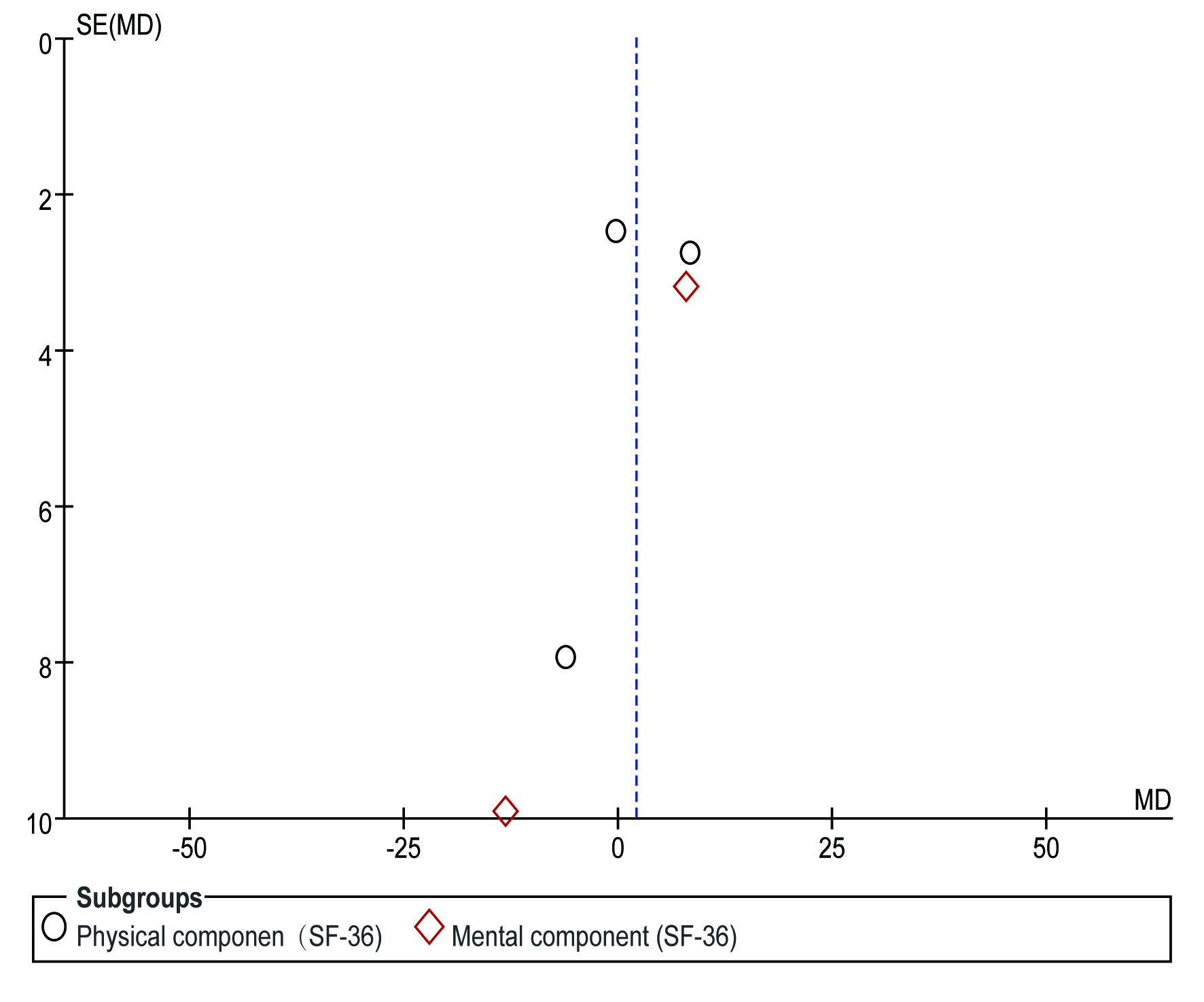

Supplement: Supplementary file 2 — Figure S1. Publication bias. (TIFF 840 kb) [file 12957_2017_1233_MOESM2_ESM.tif]
